# Human Metabolome Reference Database in a Biracial Cohort across the Adult Lifespan

**DOI:** 10.3390/metabo13050591

**Published:** 2023-04-25

**Authors:** Qu Tian, M. Gordian Adam, Enrique Ozcariz, Giovanna Fantoni, Nader M. Shehadeh, Lisa M. Turek, Victoria L. Collingham, Mary Kaileh, Ruin Moaddel, Luigi Ferrucci

**Affiliations:** 1 Translational Gerontology Branch, National Institute on Aging, Baltimore, MD 21214, USA; 2 biocrates life sciences ag, 6020 Innsbruck, Austria; 3 Laboratory of Clinical Investigation, National Institute on Aging, Baltimore, MD 21224, USA; 4 Clinical Research Unit, National Institute on Aging, Baltimore, MD 21224, USA; 5 Laboratory of Molecular Biology and Immunology, National Institute on Aging, Baltimore, MD 21224, USA

**Keywords:** aging, lifespan, reference database

## Abstract

As one of the OMICS in systems biology, metabolomics defines the metabolome and simultaneously quantifies numerous metabolites that are final or intermediate products and effectors of upstream biological processes. Metabolomics provides accurate information that helps determine the physiological steady state and biochemical changes during the aging process. To date, reference values of metabolites across the adult lifespan, especially among ethnicity groups, are lacking. The “normal” reference values according to age, sex, and race allow the characterization of whether an individual or a group deviates metabolically from normal aging, encompass a fundamental element in any study aimed at understanding mechanisms at the interface between aging and diseases. In this study, we established a metabolomics reference database from 20–100 years of age from a biracial sample of community-dwelling healthy men and women and examined metabolite associations with age, sex, and race. Reference values from well-selected healthy individuals can contribute to clinical decision-making processes of metabolic or related diseases.

## 1. Introduction

The completion of the human genome project in 2001 and the advent of post-genomics research in multi-OMICS formats, especially over the past decade, have brought human biology into a new era. As one of the OMICS in systems biology, metabolomics studies the metabolome and uses analytical techniques, such as MS and NMR, to simultaneously quantify numerous metabolites with low molecular weights (typically < 1000 Da). These metabolites are final or intermediate products and terminal effectors of upstream biological processes. Together with other areas of systems biology, such as genomics, transcriptomics, and proteomics, metabolomics provides accurate information that helps define the physiological steady state and biochemical changes during the aging process. Circulating metabolites result from a combination of genetic, epigenetic, and lifestyle factors, as well as acute and chronic exposures. Because of its close connection to phenotypes, metabolomics has been used in studies aimed at deciphering biological mechanisms that drive changes in metabolism with aging. For example, previous studies found that metabolites such as lipids, carbohydrates, carnitines, and amino acids vary substantially by age [1]. However, the previous studies had important limitations, including the fact that they included individuals that were not screened to be healthy. Therefore, differences due to aging could not be differentiated from those related to prevalent diseases or drug treatments of health conditions. In addition, the great majority of previous studies were conducted among white participants. Although there is evidence that race affects both the body’s composition and metabolism, whether metabolomic signatures of aging differ by race is unknown. Recent data have shown sex and racial differences in circulating metabolites and strongly suggest that future metabolomics studies should consider the demographic background as a stratification factor [2,3,4,5].

Studies that use metabolomics typically compare metabolites between groups of individuals or study the relationship between the metabolites and participants’ characteristics expressed by an ordinal or continuous variable. Many studies rely on relative differences because metabolomics reference data across the lifespan of men and women and different ethnic groups are not available. The availability of “normal” reference values according to age, sex, and race allows the characterization of whether an individual or a group deviates metabolically from normal aging, and these references are a fundamental element of any study aimed at understanding mechanisms at the interface between aging and diseases. A set of reference values obtained from well-selected individuals dispersed over a wide age range can potentially be used to rule out preclinical and clinical diagnoses of metabolic diseases. A few previous studies reported reference values, but they were limited to a narrow age range, focused on white participants, or included a relatively small set of metabolites [6,7,8].

In this study, we aimed to establish a metabolomics reference database across the human lifespan ranging from 20 to 100 years of age from a biracial sample of community-dwelling healthy men and women. We also aimed to examine metabolite associations with age, sex, and race.

## 2. Materials and Methods

### 2.1. Study Population

Study participants were drawn from the Baltimore Longitudinal Study of Aging (BLSA) [9] and the Genetic and Epigenetic Signatures of Translational Aging Laboratory Testing (GESTALT) [10]. The BLSA and GESTALT protocols were approved by the National Institutes of Health Institutional Review Board. All participants provided written consent at each visit. A subset of 960 participants aged between 20 to 100 years who met a pre-defined set of health status criteria was planned for this study, with comparable numbers of white men, white women, black men, and black women within each 10-year age category. Due to the limited numbers in younger and older age categories for black participants, the final study sample size was 691 (616 BLSA participants, 75 GESTALT participants) (Table 1). This analytical sample was part of a large reference database, Biocrates Quantitative Metabolomics Database (QMDB) [11].

### 2.2. Health Status

All participants in this study met the IDEAL criteria. The IDEAL status was originally designed as enrollment criteria in the BLSA and was employed weekly in the BLSA (Ferrucci 2008; Schrack et al. 2014). It was applied as enrollment criteria in the GESTALT study as well. The IDEAL criteria use both subjective and objective assessments to define the absence of health conditions and functional limitations. Specifically, participants were considered “IDEAL” if they (1) had none of the following conditions within the past 10 years, i.e., cardiovascular disease, congestive heart failure, stroke, bypass surgery, kidney disease, diabetes, and cancer; (2) had laboratory blood values within non-pathologic limits (i.e., measured systolic blood pressure of less than 145 mmHg and diastolic blood pressure of less than 90 mmHg; measured hemoglobin of 11.0 g/dL or less in men or 10.5 g/dL or less in women; measured albumin of 3.2 g/dL or less); (3) had no long-term treatment with antibiotic, antiviral, corticosteroids, immunosuppressants, or pain medications; (4) had a Blessed Information–Memory–Concentration Test score of less than 4 and a Mini-Mental State Exam of greater than 24; and (5) had no functional limitations (including no difficulty completing activities of daily living (ADLs) or independent activities of daily living (IADLs); a Short Physical Performance Battery Score of 12 if younger than 80, 11 if aged 80 to 89, or 10 if aged 90 or older; and no self-reported mobility limitations). In addition to the IDEAL status, participants included in this study also met the criteria of a body mass index of less than 35 and a usual gait speed of greater than 0.6 m/s.

### 2.3. Plasma Collection and Metabolomics Assessment

In both BLSA and GESTALT, the participants’ blood samples were collected at the NIA Clinical Research Unit, MedStar Harbor Hospital in Baltimore, Maryland. Blood samples were drawn from the antecubital vein in the morning after at least an 8-h overnight fast. Participants were not allowed to smoke, exercise, or take medications before blood samples were collected. Blood samples were collected using e–thylenediaminetetraacetic acid (EDTA) vacutainer tubes and centrifuged at 2300 rpm for 15 min, then the plasma was separated, aliquoted, and stored at −80 °C until assayed. In this study, EDTA plasma samples were collected between January 2006 and October 2018 in the BLSA and between 2015 and 2020 in the GESTALT. About 78 plasma samples were measured per plate, with up to 5 plates per week. Potential batch effects were minimized by normalizing them to target values of quality control samples present on each plate.

In both studies, metabolites were extracted, and their concentrations were measured using the MxP Quant 500 kit (Biocrates Life Sciences AG, Innsbruck, Austria) following the manufacturer’s protocol for a 5500 QTrap instrument (Sciex, Framingham, MA, USA). The approach quantifies up to 630 metabolites from 26 biochemical classes. Plasma metabolomics were assessed using liquid chromatography with tandem mass spectrometry (LC-MS/MS) for small molecules, and lipids and hexoses were measured by flow injection analysis–tandem mass spectrometry (FIA-MS/MS). The EDTA plasma samples were assayed for metabolomics between August 2019 and March 2020 in the BLSA and in December 2020 in the GESTALT. These data were uploaded to the Biocrates Quantitative Metabolomics Database (QMDB) (biocrates.com/quantitative-metabolomics-database) as a subset of a large reference database that is continuously expanded [11]. Concentration ranges and descriptive statistics data for each metabolite by age, sex, and race were automatically compiled and exported from the QMDB.2.4 Statistical analysis.

Metabolites with concentrations below the limit of detection (LOD) in more than 20% of participants were excluded from the analysis. For the remaining metabolites, values below LOD were imputed using the logspline density approach [12]. To establish the reference values of metabolites, we report the descriptive statistics of median values and interquartile ranges on the original scale across age categories in four demographic groups separately: white men, white women, black men, and black women.

To identify metabolites that were associated with age, we first log2-transformed metabolite values and examined their bivariate association with age using Pearson’s correlations in the four demographic groups separately. We also included the age squared to test for potential non-linear associations. To examine sex differences in metabolites in the overall sample, we used multivariable linear regression and adjusted for age and race. To examine racial differences in metabolites, we used multivariable linear regression and adjusted for age and sex. For these associations, we adjusted for multiple comparisons using Bonferroni correction. Significance was set and reported at Bonferroni-adjusted *p* < 0.05 (or q < 0.05). We also conducted a class enrichment analysis for metabolites associated with age, sex, and race separately using a Wilcoxon rank sum test based on the *p*-values of each metabolite [13]. We reported significantly enriched classes at *p* < 0.05.

We then conducted a Kyoto Encyclopedia of Genes and Genomes (KEGG) pathway analysis via https://www.metaboanalyst.ca/accessed on 27 February 2023. To examine pathways implicated in the age effect on metabolites, we identified the KEGG compound IDs of the metabolites significantly associated with age, including both linear and non-linear associations. Because the significance may be affected by sample sizes, we chose a fixed number of metabolites across all four groups. As 39 was the highest number of KEGG compounds associated with age in one of the four groups, a fixed number of 39 compound IDs ranked by significance were entered for the pathway analysis. To examine pathways implicated in the effect of sex or race, we entered the KEGG compound IDs for all metabolites significantly differing between the sexes or races. Note that if a metabolite was related to two KEGG compounds (for example, hexosylceramide could correspond to either glucosylceramide or galactosylceramide), both compound IDs were entered. For hexose, the compound ID for glucose was used because about 95% of the hexose in plasma is glucose. In the MetaboAnalyst pathway analysis tool, the parameters were specified as follows: scatter plot, hypergeometric test, relative-betweenness centrality, and use of all compounds in the KEGG (Homo sapiens) library. The pathway “neomycin, kanamycin, and gentamicin biosynthesis” was renamed “glucose metabolism” because hexose was the only metabolite affected in that pathway. We reported significant pathways at *p* < 0.05.

To understand whether the long-term stability affected our results, we compared the earliest (collected in 2006/2007) and most recent (collected in 2018/2019) samples that were matched by age, sex, race, and BMI using both a principal component analysis (PCA) and linear regression as sensitivity analyses.

## 3. Results

### 3.1. Reference Values

Reference values of metabolites on the original scale by age, sex, and race were exported from the QMDB and are presented in Supplementary Table S1. Up to 527 lipids (triglycerides were most represented) and up to 107 small molecules (amino-acid-related metabolites were most represented) were measured. Supplementary Table S1 lists all metabolite short names sorted by class and contains the mean and median concentrations, highest and lowest values, standard deviation, and quartiles. The percentage of samples above the LOD, the LOD thresholds, and the number of samples the values were calculated from are shown as well. After pre-processing steps, 497 metabolites remained for a further analysis. Box plots of age-, sex-, and race-associated metabolites with median, interquartile range, and individual data on the log2-transformed scale across the adult lifespan are shown in Supplementary Figure S1.

### 3.2. Metabolites Associated with Age

**Figure 1:**
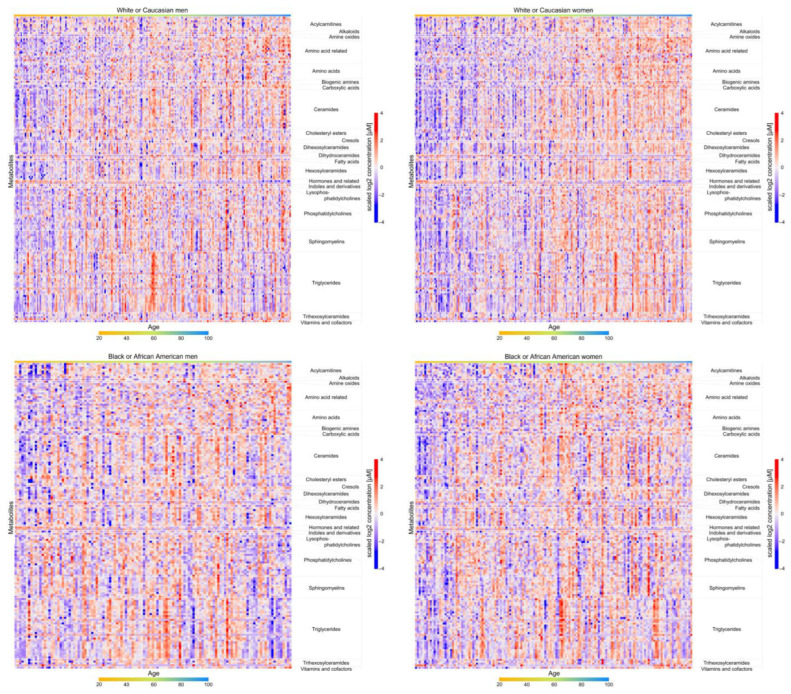
Heatmaps for metabolite associations with age by sex and race. Legend: Metabolites significantly associated with age in at least one of the four demographic groups at q < 0.05 are presented.

**Figure 2:**
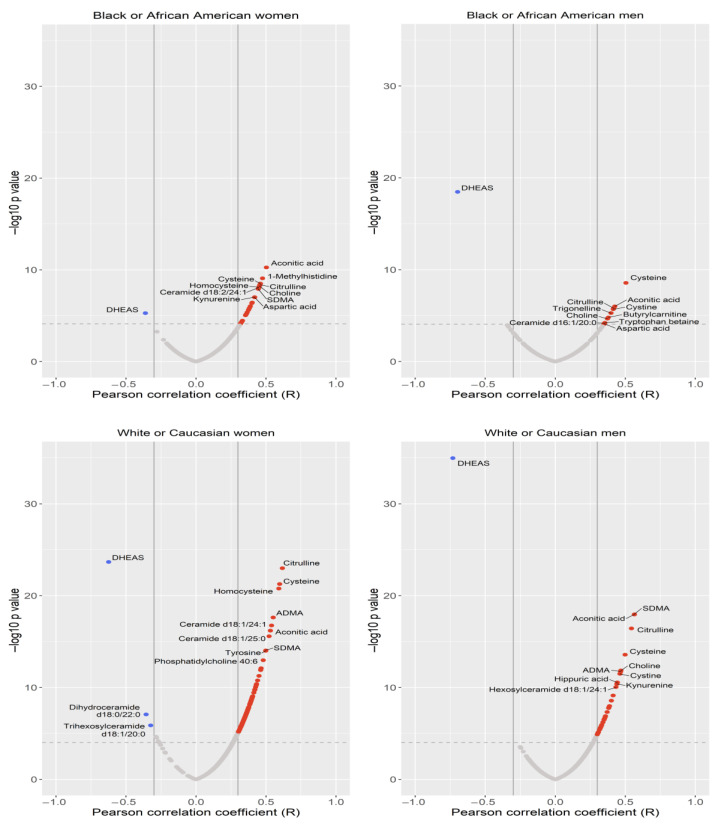
Volcano plots of metabolites associated with age by sex and race. Legend: Red indicates metabolites that are positively associated with increasing age. Blue indicates metabolites that are negatively associated with increasing age. Dashed line indicates significance level at the q-value of 0.05.

The associations between metabolites and age by sex and race are presented in Supplementary Table S2. Heatmaps for both linear and non-linear associations with age are presented in Figure 1. Volcano plots for linear associations with age are presented in Figure 2. Overall, most metabolites were positively associated with increasing age, including both linear and non-linear associations. In white men, 40 metabolites were associated with age and 39 were positively associated with age (q < 0.05); 26 metabolites showed a linear association with age and 14 showed a non-linear association. In white women, 148 metabolites were associated with age and 141 were positively associated with age (q < 0.05); 121 metabolites showed a linear association with age and 27 a non-linear association. In black men, 19 metabolites were associated with age and 15 were positively associated with age (q < 0.05); 8 metabolites showed a linear association with age and 11 showed a non-linear association. In black women, 25 metabolites were associated with age and 24 were positively associated with age (q < 0.05); 23 showed a linear association with age and 2 showed a non-linear association.

Among metabolites that were positively associated with age, seven were common in all four groups (aconitic acid, choline, citrulline, cysteine, cystine, kynurenine, and symmetric dimethylarginine (SDMA)) and 10 were common in three out of four groups (aspartic acid, asymmetric dimethylarginine (ADMA), butyrylcarnitine, ceramide d18:1/24:1, ceramide d18:1/25:0, ceramide d18:2/24:1, hippuric acid, homocysteine, methionine sulfoxide, and p-cresol sulfate) (Supplementary Table S2). One metabolite—dehydroepiandrosterone sulfate (DHEAS)—was found to be negatively associated with age across all four groups. There appeared to be more metabolites associated with age in white participants than in black participants, and some metabolites appeared to differ by sex and race. Specifically, seven metabolites were (positively) associated with age in women only, and not in men, including 1-methyhistidine, ceramide d18:1/20:0, ceramide d18:2/20:0, γ-aminobutyric acid (GABA), indoxyl sulfate, ornithine, and trimethylamine N-oxide (TMAO). Trigonelline was (positively) associated with age in black participants only and not in white participants, and there were 19 metabolites (positively) associated with age in white participants only and not in black participants.

The class enrichment analysis showed that amino-acid-related and ceramide classes were significantly enriched in all groups and acylcarnitines were enriched in three groups (white women, black men, black women), displaying a trend towards significance in the fourth group (white men) (Table 2). Carboxylic acids and trihexosylceramides were enriched in three groups except white women, and sphingomyelins were enriched in three groups except black men (Table 2). There appeared to be more enriched classes in white participants than black participants. Hexosylceramides and dihexosylceramides were enriched in white participants only and not in black participants. Phosphatidylcholines were enriched in men only and not in women.

**Figure 3:**
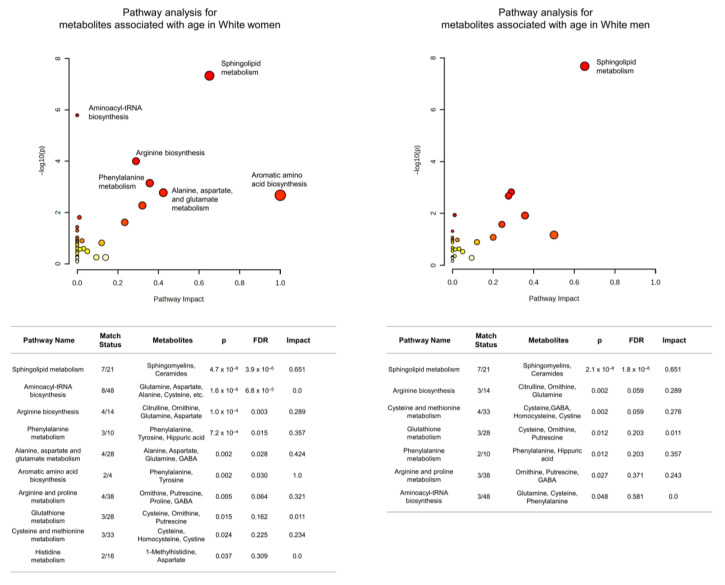

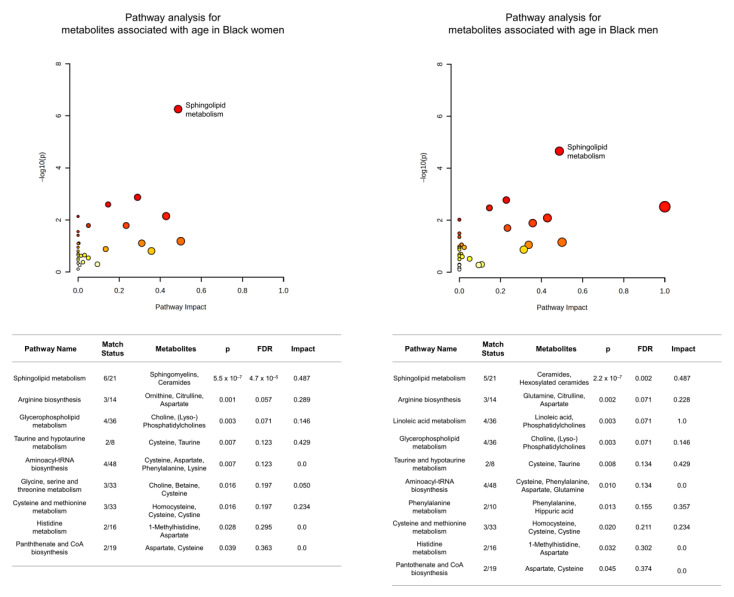
Age-associated pathways by sex and race. Legend: Pathways that survived at FDR-adjusted *p* < 0.05 are labeled with pathway names in the figure. The increase in the color scale indicates increasing significance.

The pathway analyses revealed common and different age-related pathways by sex and race (Figure 3). Four significant age-related pathways were common across the four demographic groups, including sphingolipid metabolism, arginine biosynthesis, cysteine and methionine metabolism, and aminoacyl-tRNA biosynthesis (*p* < 0.05; sphingolipid metabolism survived when FDR-adjusted to *p* < 0.05). Two significant age-related pathways were common in three out of four groups, i.e., histidine metabolism (black women, black men, white women) and phenylalanine metabolism (black men, white women, white men). Some pathways appeared to differ by race. Specifically, arginine and proline metabolism and glutathione metabolism were found significantly related to age in white participants only and not in black participants. Glycerophospholipid metabolism, taurine and hypotaurine metabolism, and pantothenate and CoA biosynthesis were found to be significantly related to age in black participants only but not in white participants.

### 3.3. Metabolite Differences by Sex

**Figure 4:**
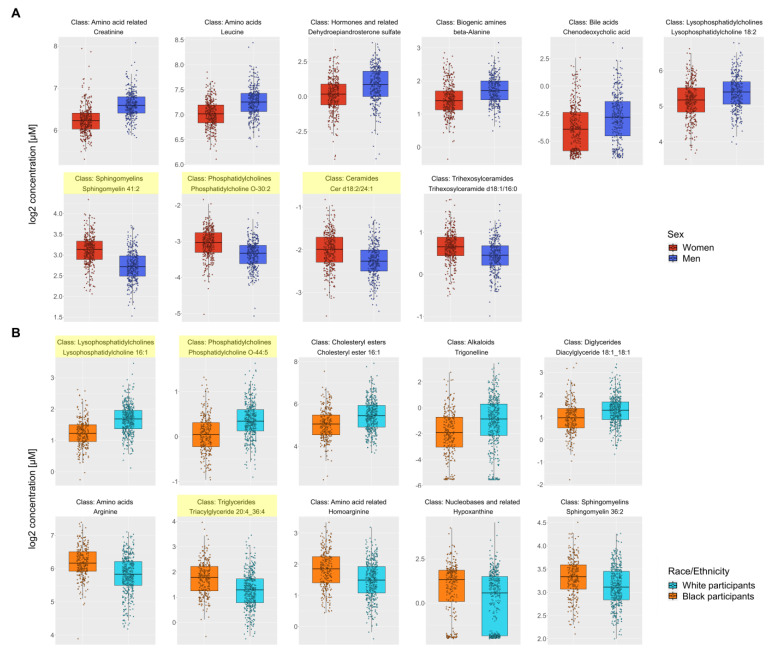
Box plots of the top significant metabolites from 10 different classes differing by sex (**A**) and race (**B**). Legend: The top row shows metabolites that were higher in men or white participants, and the bottom row shows metabolites that were higher in women or black participants. The metabolite names from significantly enriched classes are highlighted in yellow.

**Figure 5:**
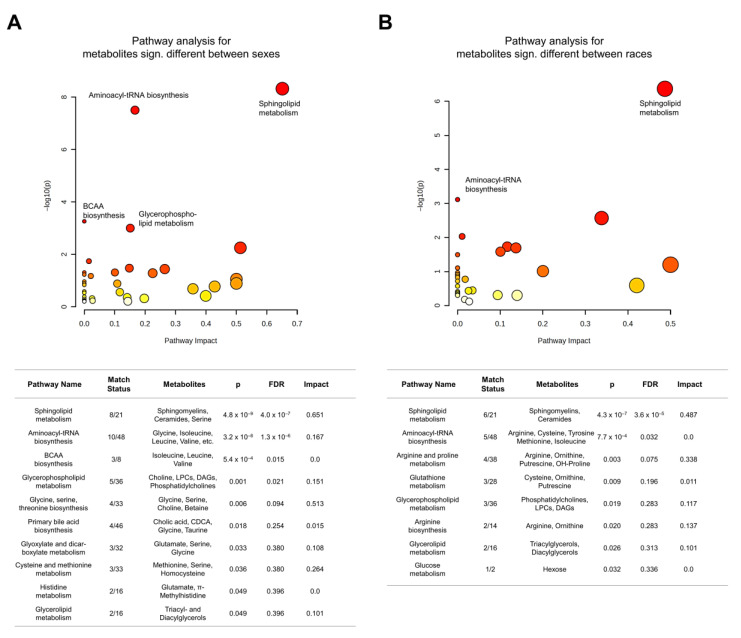
Sex- (**A**) and race-related (**B**) pathways. Legend: The increase in the color scale indicates increasing significance.

After adjustment for age and race, 123 metabolites were different by sex (all q < 0.05) (Supplementary Table S3). Four biochemical classes were significantly enriched, including phosphatidylcholines, sphingomyelins, ceramides, and dihexosylceramides (Table 3). Figure 4A shows box plots of the top significant metabolites from 10 different classes. Compared to women, men had higher creatine and leucine levels and lower sphingomyelin 41:2, phosphatidylcholine O-30:2, and ceramide d18:2/24:1 levels, which were the top significant metabolites from enriched classes. There were 10 significant pathways related to sex. Four pathways survived when FDR-adjusted to *p* < 0.05, including sphingolipid metabolism, aminoacyl-tRNA biosynthesis, BCAA biosynthesis, and glycerophospholipid metabolism (Figure 5A).

Regarding long-term stability, PCA analysis did not reveal significant differences between the earliest and most recent samples matched by demographic variables. Linear regression showed 4 metabolites were different: lactate, PC 32:3, hexose, hex3Cer d18:1/20:0 (FDR-adjusted *p* < 0.05).

### 3.4. Metabolite Differences by Race

After adjustment for age and sex, 159 metabolites were different by race (all q < 0.05) (Supplementary Table S4). Three biochemical classes were significantly enriched, i.e., lysophosphatidylcholines, phosphatidylcholines, and triglycerides (Table 3). Figure 4B shows box plots of the top significant metabolites from 10 different classes. Compared to black participants, white participants had higher lysophosphatidylcholine 16:1 and phosphatidylcholine O-44:5 levels and lower triglyceride 20:4_36:4 levels, which were the top significant metabolites from enriched classes. There were 8 pathways related to race, with sphingolipid metabolism and aminoacyl-tRNA biosynthesis surviving when FDR-adjusted to *p* < 0.05 (Figure 5B).

## 4. Discussion

Using targeted metabolomics, we established a reference metabolite database from healthy adults from 20 to 100 years of age in a biracial cohort. Data from up to 527 lipids and 107 small molecules provided information on common and unique metabolic changes with aging in men and women of white and black races. Some metabolite associations with age were expected and consistent with the literature. The novel findings of (i) common and distinct age associations by sex and race and (ii) specific sex and racial differences in metabolites add value to the existing literature.

Our metabolite reference database extends the existing knowledge. Specifically, we included metabolite values for the very young and very old adult populations across the lifespan. We also provided reference values for black and white participants, whereas previous studies primarily focused on the white population. Furthermore, the data presented here expands the QMDB, a collection of metabolite concentration ranges from healthy human individuals whose EDTA plasma samples have been measured using Biocrates kits [11]. As the QMDB allows one to customize the sample selection for concentration ranges using diverse filter options and enables users to view and export the typical concentration range for any kit metabolite, the platform makes the normative data available and facilitates the compilation of tailored reference ranges for other researchers.

Regarding lipid metabolites, studies have reported that specific metabolites are associated with increasing age (for review, see [1,14,15]), and our findings are in line with some of the previous studies. For instance, some lipid metabolites involved in lipid metabolism (such as fatty acids, carnitine, cholesterol, beta-hydroxybutyrate) and benzoate metabolism (such as hippuric acid), as well as citrulline and C-glycosyl tryptophan, have been shown to increase with age [16,17]. Creatinine was found to decrease with age [17]. Previous studies have also shown that lipid metabolites are more broadly associated with age in women than in men, and some associations differ by sex. For instance, the levels of glycerophospholipids, glycerolipids, and their subclasses (lysophosphatidylcholines, phosphatidylcholines, and triglycerides) increase more with age in women than in men, whereas the digalactosylceramide class decreases with age in women, with no age association in men [18]. The levels of phosphatidylcholines tend to increase with age in women, whereas monoacylglycerols and lysophosphatidylcholines tend to decrease with age in men [19]. The levels of sphingolipids have been observed to increase with age in women, while the association is less clear in men [4,20]. Notably, previous studies primarily examined white participants. Our findings extend prior knowledge by examining the age associations in both black and white participants. In white participants, we found consistent findings that sphingomyelins are more strongly associated with age in women than men. We also found that the association of sphingomyelins with age differed by race. The association was stronger in white participants than in black participants.

Our results on the associations between non-lipid metabolites with age also share consistencies with previous findings. For instance, citrulline and SDMA have been shown to increase with age [17], possibly due to deficits in urea cycle efficiency and declining renal function with age [21,22]. Cysteine and the related dimer cystine have also been shown to increase with age, possibly attributed to declining renal function [23] or a decreased capacity for glutathione synthesis [24]. A positive correlation of aconitic acid levels with age has been reported [4,16], and this age-related increase may be due to declining mitochondrial function. An age-related increase in kynurenine was also reported previously and may be caused by elevated low-grade inflammation promoting the synthesis of kynurenine and its downstream metabolites from the amino acid tryptophan [25]. In addition, we observed that plasma choline was positively associated with age, a finding that has yet to be reported. High levels of circulating choline along with low levels of betaine were associated with cardiovascular risk [26]. As choline undergoes mitochondrial oxidation to betaine, high levels of choline and low levels of betaine may suggest impaired mitochondrial choline oxidation. Notably, most of the metabolites, including lipids and non-lipids, were found to be positively associated with age in our study. One possible contributing factor may be the decreasing water content in the blood with age [27], leading broadly to higher concentrations. The hormone DHEAS was the only metabolite negatively associated with age. The age-dependent decline in the adrenal production of DHEAS is known, although the underlying causes are still unclear [28].

Our findings on sex differences in metabolites are consistent with previous studies. Lipid classes (including sphingomyelins) are generally higher in women than men, whereas biogenic amines, amino acids, and acylcarnitines tend to be higher in men [6,7]. Men had higher concentrations of creatinine, leucine, and isoleucine than women. Because men have greater muscle mass and strength than women, these findings support previously reported associations between creatinine and muscle mass [29] and between leucine and isoleucine and muscle strength [30]. Women showed higher cortisol levels collected in the morning than men, a finding that is consistent with the literature [31]. Previous pathway analyses have found that metabolites involved in oxidative phosphorylation, sphingolipids, and long-chain fatty acids are higher in women, whereas branched-chain amino acids, bile acids, and steroids are higher in men [32]. The findings on sex differences of lysophosphatidylcholines are somewhat mixed; some reported that men had higher concentrations of lysophosphatidylcholines than women, whereas others showed that women had higher concentrations [7].

Data on metabolite differences by race among healthy individuals are sparse. To the best of our knowledge, only one study examined racial differences using targeted metabolomics in women only. They found that most lipid levels were lower in black women than white women [33] and enriched classes included glycerolipids, glycerophospholipids, and sterol lipids. We found consistent race-dependent enrichments in glycerophospholipids (lysophosphatidylcholines and phosphatidylcholines) and glycerolipids (triglycerides). In our study, the sterol lipids (cholesteryl esters) class was ranked 4th among the enriched classes, although it was not statistically significant. Our findings of the top significant metabolites confirmed and extended prior findings. In both studies, black participants had higher levels than white participants of arginine, homoarginine, hydroxyproline, and creatinine and lower non-lipid metabolites, i.e., ornithine, kynurenine, and trigonelline. We also found that black participants had higher methionine and lower tryptophan betaine and putrescine levels than white participants. It remains unclear what factors explain these observed racial differences but likely involve lifestyle, diet, genetics, environment, and socio-economic status factors. Future studies with larger sample sizes and a more diverse group of participants are needed to confirm these racial differences and understand the underlying mechanisms.

This study had limitations. The sample size was modest, especially the low number of black participants in the 20s and 90s age groups, with only one black man in the 90+ group, a factor that may have affected the statistical significance of some findings. Additionally, since metabolites can rapidly change in response to environmental conditions, such as the consumption of certain foods, exposure to drugs, and changes in the microbiome induced by antibiotics, the current normative data should be interpreted with caution. Although our analyses did not reveal a substantial long-term stability effect, the plasma storage time reportedly affects metabolite levels, especially lipids [34,35,36]. This study also had several strengths. First, the reference database encompassed a wide age range of 20 to 100 years of age to capture the adult lifespan. Second, the database included two races and included both men and women, representing a comprehensive demography. Third, the demographic groups allowed us to investigate how aging affects the human metabolome by sex and race. Fourth, the study population was well characterized and met the stringent criteria for a “healthy” or “ideal” status, minimizing the influence of preclinical and clinical diseases on this database. Well-defined metabolite concentration ranges for healthy individuals, as presented in this study, which is part of the QMDB, can serve as reference values, and in the long run contribute to clinical decision-making processes.

## 5. Conclusions

In conclusion, our reference database, which includes up to 527 lipids and 107 small molecules, of the human metabolome is broadly applicable and underscores unique aspects of a wide age range and two races, white and black. Among the four demographic groups, metabolite profiles across the lifespan share similarities and also show distinct patterns. Although our findings provided unique normative data that allowed an initial comparison between black and white participants, our sample size was relatively small, and our findings should be independently validated in a large population.

## Figures and Tables

**Table 1:** Participants’ characteristics.

Age	20–30	30–40	40–50	50–60	60–70	70–80	80–90	90+
	Sample size, n							
White Men (n = 204)	10	29	24	30	30	30	30	21
White Women (n = 213)	13	30	28	30	30	30	30	22
Black Men (n = 122)	4	7	14	30	30	23	14	1
Black Women (n = 148)	3	5	22	30	30	30	28	3
	Age, Mean ± SD (range)							
White Men	25.2 ± 2.5 (22–29)	34.8 ± 3.1 (30–39)	46.9 ± 2.1 (41–49)	55.7 ± 2.9 (50–59)	65.5 ± 2.4 (60–69)	74.0 ± 2.7 (70–79)	83.7 ± 2.5 (80–89)	91.4 ± 1.4 (90–94)
White Women	27.0 ± 1.6 (24–29)	36.1 ± 2.7 (30–39)	45.5 ± 2.4 (40–49)	56.2 ± 3.0 (50–59)	65.6 ± 2.6 (60–69)	73.4 ± 2.3 (70–78)	83.4 ± 2.3 (80–88)	90.8 ± 1.3 (90–94)
Black Men	26.5 ± 1.0 (26–28)	33.4 ± 3.4 (31–39)	45.7 ± 2.3 (40–49)	54.5 ± 2.6 (50–59)	64.4±2.7 (61–69)	73.2 ± 2.5 (70–78)	81.8 ± 2.0 (80–87)	91 –
Black Women	28 ± 0 28	35.8 ± 1.6 (34–38)	46.1 ± 2.5 (40–49)	56.3 ± 2.8 (51–59)	65.3 ± 3.2 (60–69)	73.1 ± 2.5 (70–79)	83.0 ± 2.0 (80–87)	91.7 ± 2.1 (90–94)
	Body mass index, kg/m^2^, Mean ± SD							
White Men	25.0 ± 2.4	25.9 ± 3.1	27.4 ± 3.7	27.1 ± 3.3	28.7 ± 3.3	26.6 ± 2.5	25.8 ± 3.1	25.8 ± 3.6
White Women	25.4 ± 3.0	24.1 ± 3.8	24.6 ± 3.7	24.9 ± 3.7	26.1 ± 4.8	25.6 ± 3.6	24.8 ± 3.3	23.8 ± 3.7
Black Men	25.1 ± 3.6	24.7 ± 1.7	28.0 ± 3.8	29.0 ± 3.9	28.4 ± 3.6	28.3 ± 3.8	27.0 ± 3.9	24.7
Black Women	23.6 ± 2.9	28.6 ± 3.4	26.1 ± 3.8	26.7 ± 3.4	29.1 ± 3.7	28.6 ± 3.7	26.5 ± 3.6	26.7 ± 3.3
	Gait speed, m/s, Mean ± SD							
White Men	1.33 ± 0.15	1.37 ± 0.23	1.38 ± 0.16	1.35 ± 0.19	1.31 ± 0.19	1.26 ± 0.17	1.17 ± 0.15	0.95 ± 0.14
White Women	1.35 ± 0.15	1.36 ± 0.19	1.39 ± 0.17	1.33 ± 0.18	1.31 ± 0.15	1.17 ± 0.16	1.14 ± 0.14	0.92 ± 0.17
Black Men	1.32 ± 0.36	1.20 ± 0.14	1.30 ± 0.30	1.20 ± 0.18	1.16 ± 0.15	1.18 ± 0.25	1.08 ± 0.18	1.12
Black Women	1.24 ± 0.08	1.42 ± 0.30	1.19 ± 0.19	1.17 ± 0.15	1.09 ± 0.11	1.04 ± 0.18	0.96 ± 0.13	0.80 ± 0.27

**Table 2:** The class enrichment analysis results for metabolites associated with age by sex and race.

	White Men	White Women	Black Men	Black Women
Biochemical Classes	*p*-Value			
Acylcarnitines	0.057	**0.007**	**0.004**	**3.9 × 10^−6^**
Amino-acid-related	**0.001**	**0.001**	**3.2 × 10^−5^**	**1.2 × 10^−9^**
Amino acids	0.115	0.332	0.298	0.307
Bile acids	0.985	0.991	0.197	0.983
Biogenic amines	0.284	0.421	0.121	0.106
Carboxylic acids	**0.041**	0.132	**0.042**	**0.022**
Ceramides	**3.8 × 10^−4^**	**2.0 × 10^−8^**	**0.009**	**2.8 × 10^−6^**
Cholesteryl esters	0.812	0.678	**0.002**	0.914
Diglycerides	0.612	0.999	0.676	0.763
Dihexosylceramides	**3.4 × 10^−4^**	**0.004**	0.138	0.269
Dihydroceramides	0.181	**0.047**	0.635	0.422
Fatty acids	0.163	0.971	0.518	0.508
Hexosylceramides	**1.7 × 10^−7^**	**0.003**	0.886	0.393
Hormones and related	0.119	0.228	0.097	0.063
Indoles and derivatives	0.541	0.859	0.439	0.083
Lysophosphatidylcholines	0.897	0.990	0.896	0.591
Phosphatidylcholines	**3.3 × 10^−7^**	0.859	**0.001**	0.865
Sphingomyelins	**0.020**	**2.7 × 10^−5^**	0.897	**0.014**
Triglycerides	0.999	0.999	0.999	0.999
Trihexosylceramides	**0.004**	0.120	**0.013**	**0.014**

Footnotes: Bold numbers indicate significantly enriched classes at *p* < 0.05.

**Table 3:** The enrichment analysis results for metabolites associated with sex and race.

Classes Associated with Sex	*p*-Value	Classes Associated with Race	*p*-Value
Phosphatidylcholines	**2.2 × 10^−11^**	Lysophosphatidylcholines	**0.045**
Sphingomyelins	**2.9 × 10^−8^**	Phosphatidylcholines	**0.046**
Ceramides	**4.2 × 10^−4^**	Triglycerides	**0.047**
Dihexosylceramides	**0.047**	Cholesteryl esters	0.204
Trihexosylceramides	0.051	Dihexosylceramides	0.275
Amino acids	0.064	Diglycerides	0.315
Amino-acid-related	0.077	Amino-acid-related	0.326
Hexosylceramides	0.079	Sphingomyelins	0.393
Cholesteryl esters	0.081	Bile acids	0.439
Hormones and related	0.119	Dihydroceramides	0.454
Diglycerides	0.305	Indoles and derivatives	0.586
Biogenic amines	0.417	Hormones and related	0.742
Acylcarnitines	0.590	Biogenic amines	0.749
Dihydroceramides	0.725	Amino acids	0.813
Lysophosphatidylchlines	0.862	Fatty acids	0.829
Bile acids	0.885	Trihexosylceramides	0.882
Indoles and derivatives	0.944	Acylcarnitines	0.908
Fatty acids	0.972	Carboxylic acids	0.970
Carboxylic acids	0.997	Hexosylceramides	0.998
Triglycerides	0.999	Ceramides	0.999

Footnote: Bold number indicates significantly enriched classes at *p* < 0.05. Classes ranked by significance.

## Data Availability

Publicly available datasets were analyzed in this study. This data can be available upon request by proposal submission through the Baltimore Longitudinal Study of Aging website (www.blsa.nih.gov, accessed on 27 February 2023). All requests are reviewed by the Data Sharing Proposal Committee and are also subject to approval from the NIH Institutional Review Board.

## Supplementary Materials

The following supporting information can be downloaded at: https://www.mdpi.com/article/10.3390/metabo13050591/s1. Figure S1: Box plots of metabolites across the adult lifespan by sex and race. Table S1: Descriptive statistics on metabolites across the adult lifespan by sex and race. Table S2: Associations between metabolites and age by sex and race. Table S3: Age- and race-adjusted associations between metabolites and sex. Table S4: Age- and sex-adjusted associations between metabolites and race.
