# Supplementary figures and images for: Human Metabolome Reference Database in a Biracial Cohort across the Adult Lifespan
Source: Metabolites. 2023 Apr 25;13(5):591. doi: 10.3390/metabo13050591 (PMC10221446; doi:10.3390/metabo13050591)

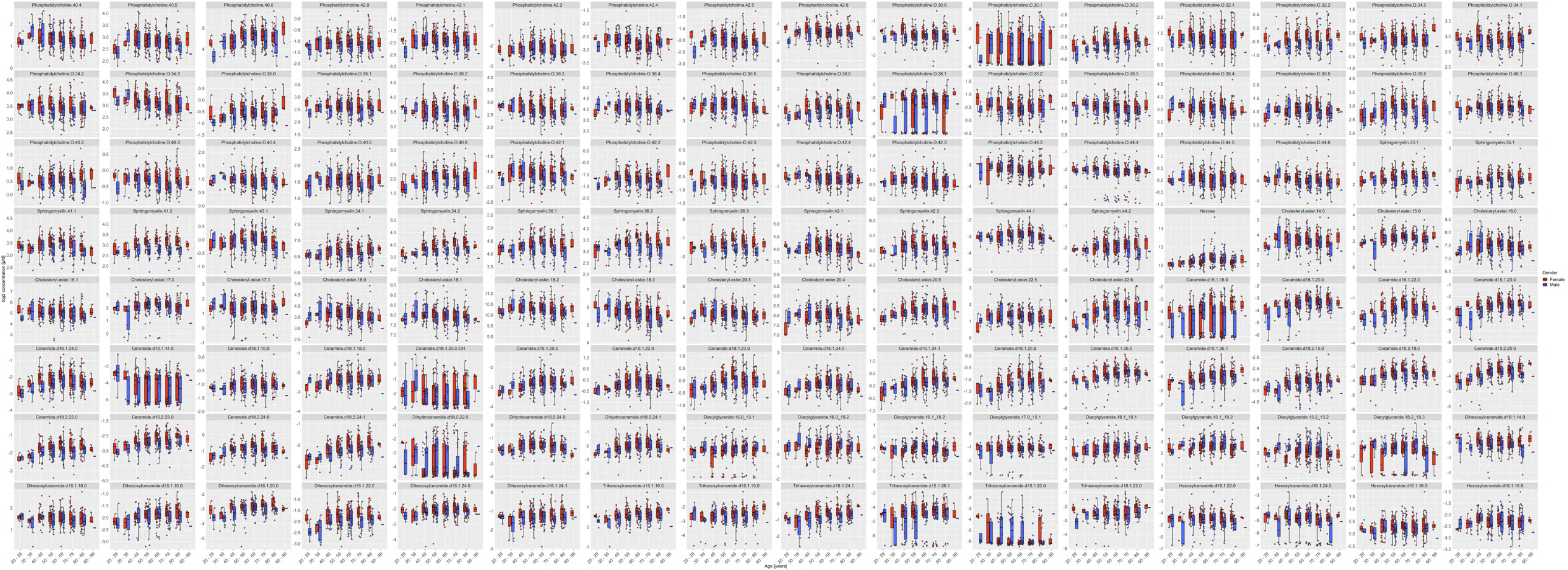

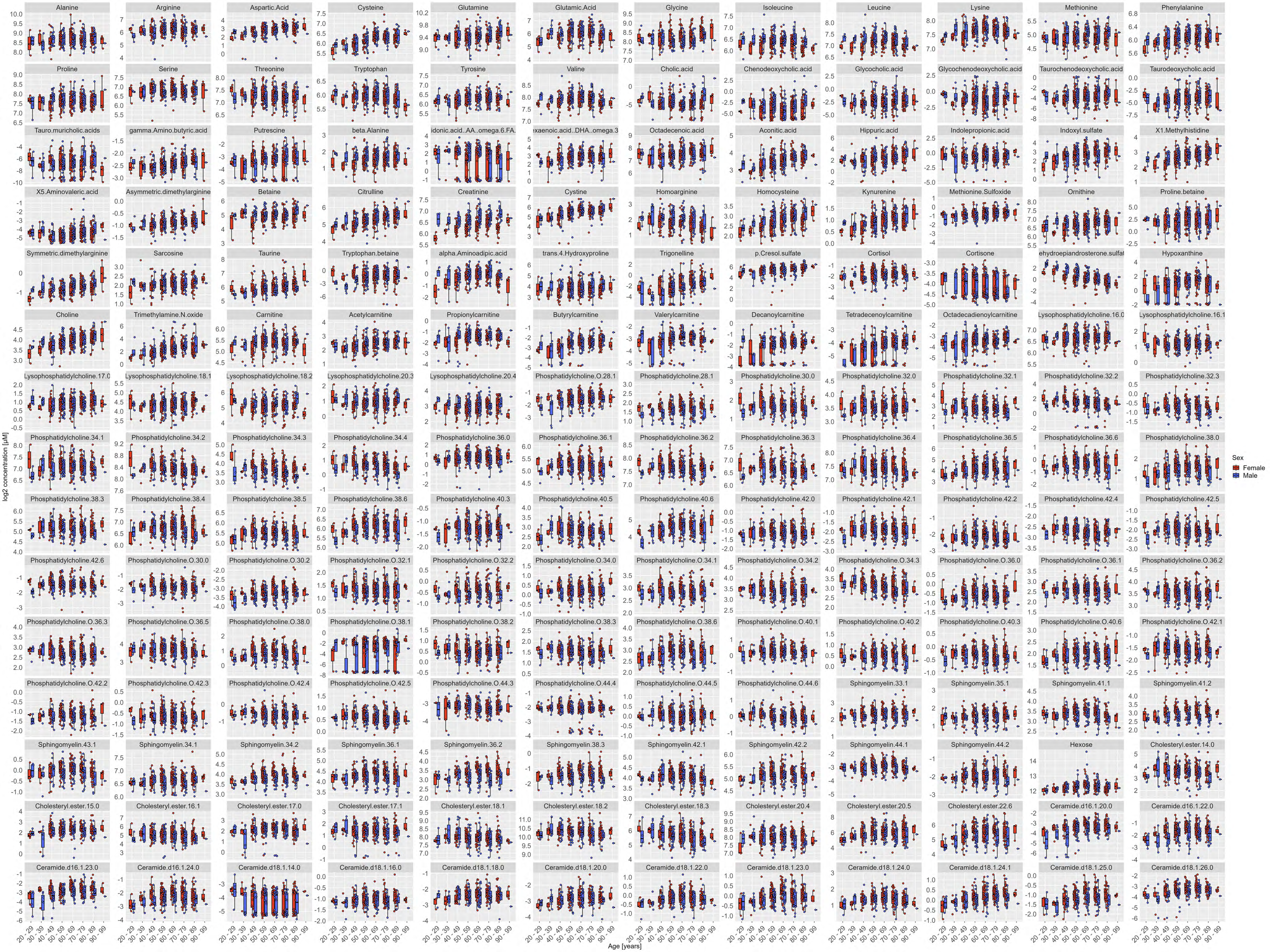

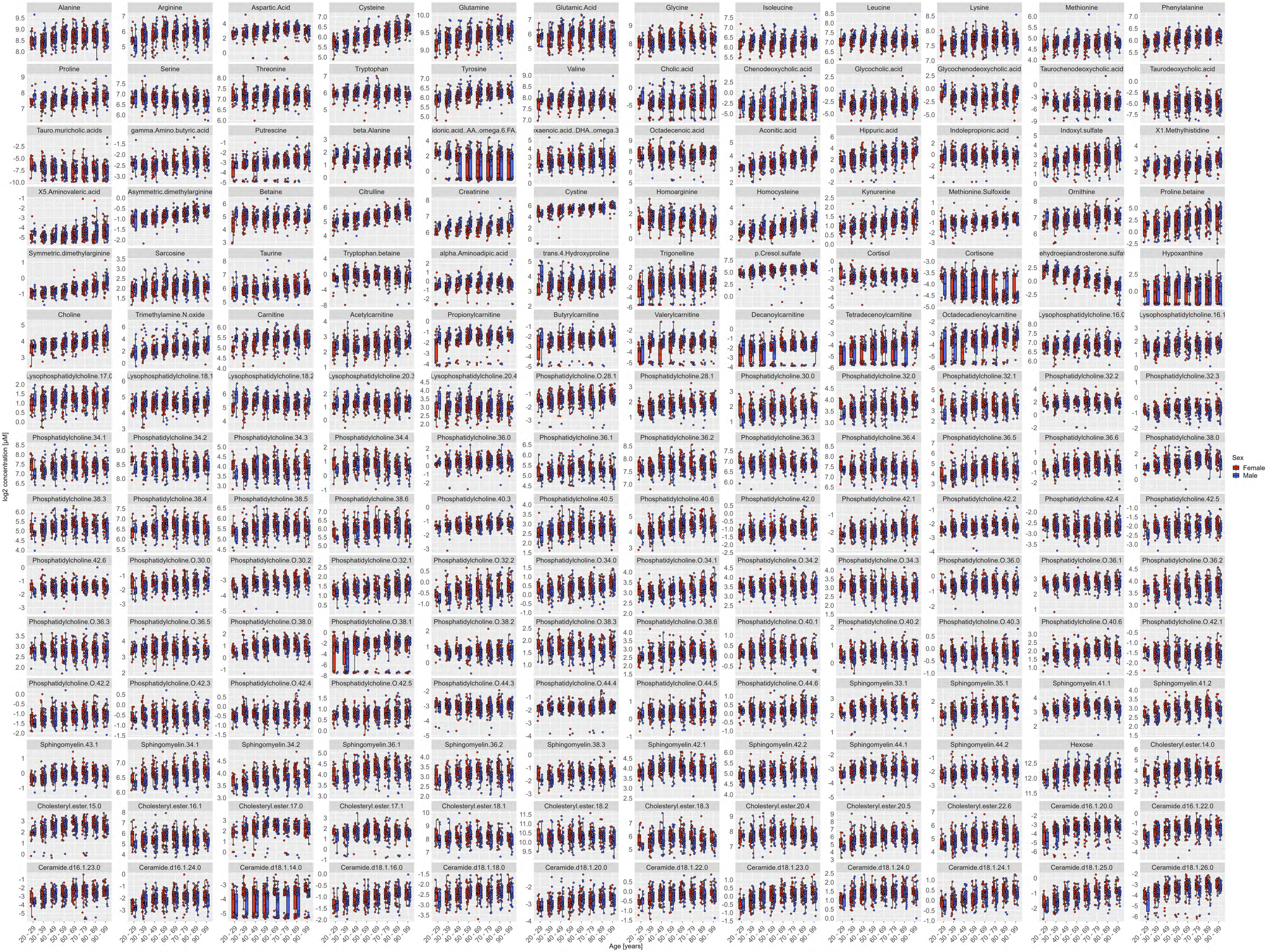

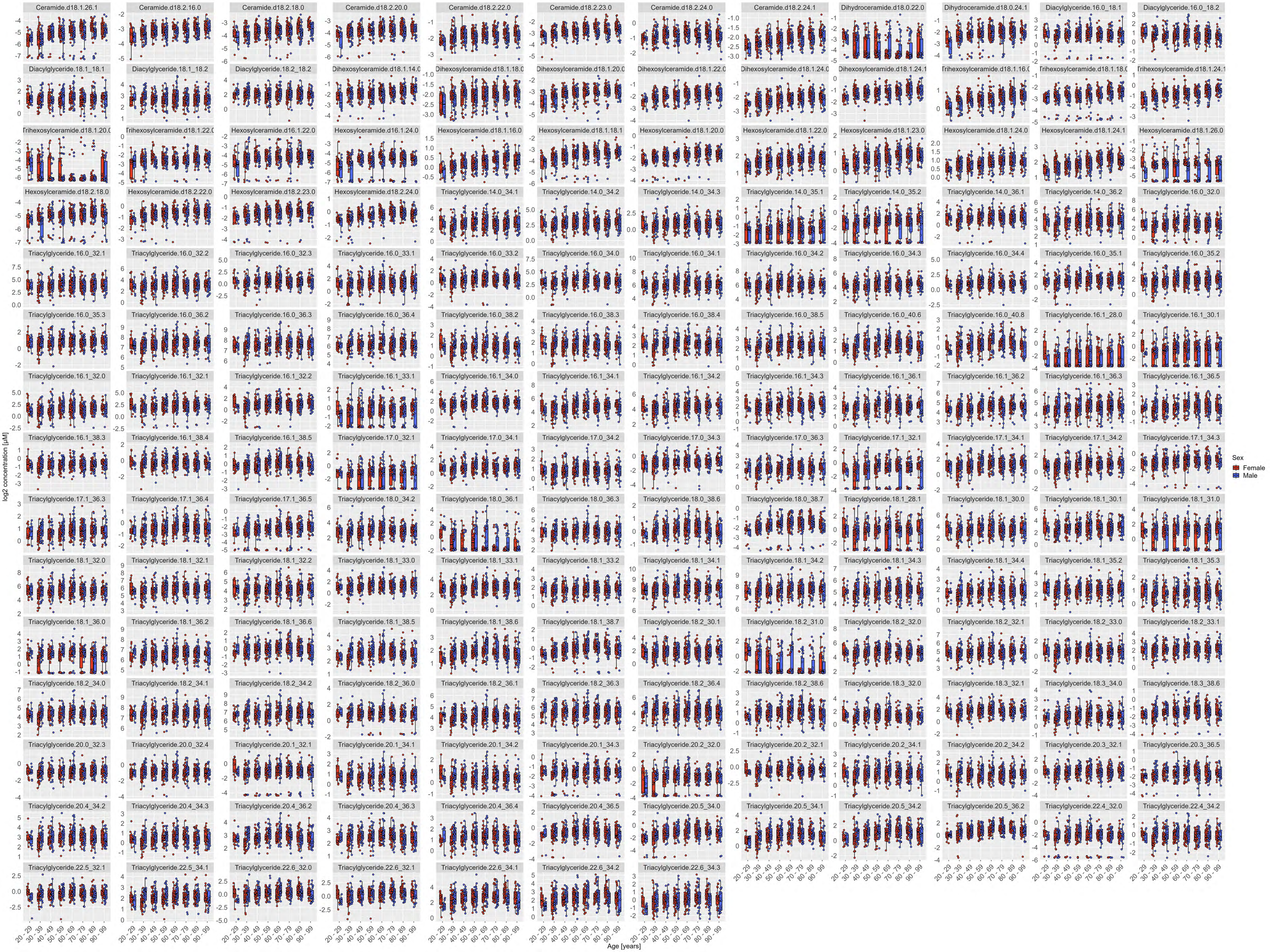

Supplement: Supplementary file 1 [file metabolites-13-00591-s001.zip › Supplementary Figure S1.pdf]
